# The role of myeloid-derived suppressor cells in liver cancer

**DOI:** 10.1007/s12672-023-00681-8

**Published:** 2023-05-23

**Authors:** Shiyue Zhou, Zixuan Zhao, Hao Zhong, Zehao Ren, Yuye Li, Hong Wang, Yuling Qiu

**Affiliations:** 1 State Key Laboratory of Component-based Chinese Medicine, Tianjin University of Traditional Chinese Medicine, 10 Poyanghu Road, Jinghai District, Tianjin, 301617 People’s Republic of China; 2 School of Medical Technology, Tianjin University of Traditional Chinese Medicine, 10 Poyanghu Rd., West Area, Tuanbo New Town, Jinghai Dist, Tianjin, 301617 China; 3 Binhai New Area Hospital of TCM, Tianjin, 300451 China; 4 School of Pharmacy, Tianjin Medical University, Tianjin, 300070 China

## Abstract

MDSCs are immature myeloid immune cells, which accumulate in models of liver cancer to reduce effector immune cell activity, contribute to immune escape and treatment resistance. The accumulation of MDSCs suppresses the role of CTL and the killing effects of NK cells, induces the accumulation of Treg cells, and blocks the antigen presentation of DCs, thus promoting the progression of liver cancer. Recently, immunotherapy has emerged a valuable approach following chemoradiotherapy in the therapy of advanced liver cancer. A considerable increasing of researches had proved that targeting MDSCs has become one of the therapeutic targets to enhance tumor immunity. In preclinical study models, targeting MDSCs have shown encouraging results in both alone and in combination administration. In this paper, we elaborated immune microenvironment of the liver, function and regulatory mechanisms of MDSCs, and therapeutic approaches to target MDSCs. We also expect these strategies to supply new views for future immunotherapy for the treatment of liver cancer.

## Introduction

Liver cancer is the third most common reason of cancer-associated fatalities globally next only to stomach and lung cancer [1–4]. HCC is the major kind of liver cancer [5, 6], the ratio roughly 75% of cases [7], while ICC accounts for about 10% [8–10], liver cancer also includes other rare tumors including fibrous lamellar carcinoma and hepatoblastoma [11]. The prevalence of liver cancer has stabilized currently compared to other cancers, but the relative 5-year survival rate for HCC still low at 18% [12, 13]. Patients can be treated at earlier stage hepatoma by surgical, local ablation or liver transplantation, with a three-year survival rate of 30–45% [14]. There was still inadequacy of therapeutic approach for advanced liver cancer that cannot be treated surgically or primary HCC with distant metastases [15]. Therapeutic resistance and toxicity remain to be major challenges for HCC first-line drugs, such as Sorafenib and Lenvatinib. In recent years, it is well known that immunotherapy, especially immune checkpoints, has shown significant therapeutic effect in some cancers [16, 17].

The unsatisfactory results of cancer treatment have been partly explained by immune escape owing to the inability of cancer cell antigens to be recognized by immune cells and the development of immunosuppressive microenvironment [18]. This may be partly due to liver cancer’s tendency to release chemokine [19] and evade immune surveillance by activating and recruiting immunosuppressive cells like MDSCs, Treg, and TAMs, leading to immunosuppression and accelerated metastatic tendencies. There are numerous immunosuppressive cells in the TME, which take essential part in the process of tumor formation as well as immune escape [20, 21]. Immunotherapy is on the ground of the Physical body own immune system, which can improve the body's ability to kill tumor cells actively or passively through cytokine regulation, relay cell transfusion back, tumor cells and their subcellular tumor antigen components vaccine, DC vaccine, etc., in order to achieve the goal of cancer treatment [22].

In the last few years, research on the effect of MDSCs has further deepened our understanding of oncology [23, 24]. The characteristics of MDSCs inhibit the function of immune cells in the body, so that cancer cells avoid detection and attack by host immune system [25, 26]. Moreover, they encourage the survival of cancer cells by inducing a number of factors, like stimulating angiogenesis [27] and formation of pre-metastasis microenvironment as well as other non-immune mechanisms to stimulate the growth of tumors [28]. In patients with tumors and tumor-bearing mice, MDSCs levels are closely related to poor prognosis as well as treatment outcomes. At present, targeting MDSCs therapy has become one of the therapeutic strategies for liver cancer [29].

This review describes the potential association of MDSCs in the TME with HCC progression. Further, the review summarized the links between MDSCs and other immune cells, regulation of MDSCs signaling pathways, and targeted therapy of MDSCs. Finally, we discussed the prospects and challenges of preclinical research on MDSCs.

## Characteristics of liver microenvironment formation

Liver is the largest metabolic and detoxification organ in physical body, as well as a significant immune organ of the body, with roles of metabolism, detoxification, biotransformation, synthesis and immunity [30–32]. At the same time, the liver has a dual blood source system that includes hepatic arterial system and portal venous system. This portal vein provides about 70–80% of the blood to the liver, which can transfer microorganism’s bacteria and carrying nutrients. Meanwhile, the liver needs to provide immune detection for the stability of the body while removing harmful antigens [33, 34]. In order to avoid liver injury caused by immune system overreaction, the hepar has formed a unique immune tolerance microenvironment. Clinical data show that patients in transplantation of the liver without or with lower levels of immunosuppressive agents are able to establish immune tolerance [35]. This unique phenomenon of immune tolerance is closely associated with the functions performed by the immune cells.

## Mechanism of hepatic immunosuppressive microenvironment

Plenty of immune-related cells are existed in the liver, inclusive of APC, lymphocytes as well as myeloid cells, which regulate the immune response of the hepar. The APCs in the hepar include hepatic LSEC, hepatic macrophages or KC, and DCs [36]. The presence of these cells forms the liver's first line of safeguard the external pathogen invasion as well as antigenic substances, and can eliminate harmful substances through endocytosis without affecting the absorption of nutrients. In contrast to other immune organs, the phagocytic clearance of antigens by APCs in the liver normally limits the activation of adaptive immunity and inhibits the differentiation as well as maturation of effector cells, promoting the proliferation of regulatory cell subsets [37, 38]. Lymphocytes in the liver are usually located in the portal vein, but some are also scattered among the liver parenchyma and inclusive a great deal of immune effector cells. Among them, Foxp3 Treg, which are turning into CD4^+^ T cells, can inhibit the secretion of granzyme B in CD8^+^ T cells and thus impair the effect of CTL [39–41]. The main mechanisms of immune suppression by Treg cells include: compromising T-cell function by expressing IL-2 receptors that plunder IL-2 from the microenvironment in large numbers; expressing CTLA-4, which binds to and downregulates CD80/CD86 costimulatory molecules selectively on APCs to prevent T cells from receiving costimulatory signals; and generating immunosuppressive cytokines, for example, IL-10 and TGF-β that affect CTL function by forming extracellular adenosine from ATP via CD39 and CD73 to bind to the corresponding receptors on T cells [42]. Chemokine ligand 20 (CCL-20) is a cytokine that recruits Treg, and liver cancer enhances Treg activity through a transcriptional network comprising the cytokines CCL20-IL17-IL6 to promote tumor cell escape and metastasis [43]. In addition, Treg cells influence the normal effect of NK cells by selectively expressing TGF-β [45]. NK cells are an important category of immune cells that can engage with tumor cells through the release of perforin or granzyme to lyse cancer cells [44–46]. However, when the TME is hypoxic, it causes NK cells to consistently activate the target of rapamycin-GTPase kinesin-related protein 1, leading to damaged mitochondria, reduced NK numbers and cytotoxicity, and ultimately escape of HCC cells [47]. T cells are key cells in cellular immunity and are able to exert killing effects on tumors by secreting enzymes, cytokines, etc. However, in some cases such as HCV chronic infection, T cells are intrinsically regulated by increasing the level of immune checkpoints including PD-1 or CTLA-4, that lead to CD8^+^ T cell dysfunction as well as difficulty in suppressing the development of tumors [48].

Myeloid cells, inclusive of macrophages, DCs, neutrophils, as well as MDSCs, are the most plentiful immune cells and are essential for maintaining hepatic immunological homeostasis. Among them, neutrophils, as the most prevalent immune cells in the body, is a significant element of the immunosuppressive microenvironment of HCC and are closely related to the development of HCC, immune escape and drug sensitivity. Neutrophils were classified into two phenotypes, the N1 phenotype and the N2 phenotype. Neutrophils of the N2 phenotype promote tumor cell growth and migration by releasing pro-tumor growth factors and remodeling the extracellular matrix [49]. In addition, they can promote angiogenesis of tumor cells and suppress the effect of CTLs and NK cells [50]. MDSCs are a diverse subset of cells that have significant immunosuppressive properties, and various factors (e.g., tumor-associated inflammation, angiogenic factors, and chemokine gradients) coordinate the expansion as well as accumulation of MDSCs. An increasing of MDSCs is related to poor survival in the tumor-bearing host. The level of Arg-1, the generation of NO as well as ROS were all elevated by the activation of MDSCs in malignancies [51]. It is TLR2 ligands and T cell-derived IFN-γ that enhanced M-MDSC-mediated immunosuppression [52]. Through these mechanisms, MDSCs are trapped in the formation of immunosuppressive microenvironment of liver tumors as well as accelerate the progression of HCC.

## The origin of MDSCs and the mechanism of mediated immunosuppression

MDSCs are highly heterogeneous immature myeloid cells (IMCs) that originate from HSCs. HSCs can develop into myeloid progenitor cells when the environment is healthy, which can eventually differentiate into DC, macrophages and neutrophils [53–55]. In healthy individual, MDSCs are barely detectable (IMCs are detected according to the protein marker CD11b+ Gr-1+ in mice). Nevertheless, under pathological conditions, like infection, trauma and especially in the case of tumors, sustained stimulation often results in defective differentiation of IMCs, and a large number of cells in a non-maturing state accumulate in multiple organs, which stimulated by some inflammatory factors, eventually induce the formation of MDSCs [56]. MDSCs are accumulated into peripheral blood, BM, spleen, lung, liver and tumor, especially MDSCs in the spleen, which is 40% taller.

In mice and humans, MDSCs can be divided into PMN-MDSC and M-MDSC based on their density, morphology as well as phenotype [57]. The PMN-MDSC are regarded as CD15^+^CD14^−^CD33^dim^HLA-DR^neg^ or, and M-MDSC as CD15^−^CD14 ^+^CD33^pos^HLA-DR^neg^ in human [58, 59]. In addition, another immature cell subpopulation in humans defined as lacking spectral markers, inclusive of CD3, CD14, CD15, CD19, CD56, HLA-DR, as well as expressing CD33, was defined as early MDSCs. It is similarly that neutrophils and PMN-MDSC in phenotype and function, make it difficult to distinguish between the two. Dmitry Gabrilovich's group confirmed that LOX-1 is a specific marker to recognize neutrophils and PMN-MDSC of humans [60, 61]. In tumor-bearing mice, MDSCs is described by markers different from those used in humans. The subpopulations of MDSCs can be distinguished by identifying CD11b and Gr-1 [62]. These markers can be used to separate MDSCs groups more accurately. In tumor-bearing mice, PMN-MDSC can be regarded as CD11b^+^Ly6G^+^Ly6C^lo^ and M-MDSC as CD11b^+^Ly6G^−^Ly6C^hi^, with other markers under investigation [63]. The main characteristic of MDSCs is immunosuppression and play an immune role through numerous mechanisms of cancer [64, 65]. The function, distribution and immunosuppressive mechanisms of M-MDSC cells are different from PMN-MDSC cells. In general, PMN-MDSC are very common in the TME, while M-MDSCs mainly accumulate in peripheral blood, which promotes tumor growth and metastasis in various ways [66]. M-MDSC produces stronger immunosuppressive activity than PMN-MDSC due to the fact that M-MDSC expresses Arg-1 and produces IL-10, TGF-β and NO, while PMN-MDSC produces more ROS (present for a shorter period of time). The role of MDSCs immunosuppressive functions can involve in various pathways, such as increase production of Arg1, iNOS, ROS, and nitrogen substances like peroxynitrite (PNT), and other immunosuppressive factors. L-arginine, an important amino acid necessary for T cell proliferation, is depleted and converted to urea and L-ornithine when Arg-1 is upregulated in MDSCs [67]. Under L-arginine restriction, the up-regulation of iNOS in MDSCs results in the generation of NO, which interacts with superoxide to generate PNT [68]. The production of PNT by MDSCs leads to nitration and nitrosoylation of TCR, which disrupts the role of CD8^+^ T and leads to T cell tolerance [69, 70]. In addition, PNT lead to nitration chemokines, such as CCL2, and reducing the interacting of antigenic peptides and cancer cell, and reducing infiltration of CD8^+^ T cells. Therefore, elucidating the key mechanisms of recruitment and MDSC’s invasion into the TME is an important method to block MDSCs from reversing the immunosuppressive TME.

Moreover, factors in TME such as GM-CSF, G-CSF, SCF or S100A8/9 released from tumor cells can recruit MDSCs for proliferation via STAT3 and c/EPBβ [71]. MDSCs recruitment and expanding population, and suppressing body’s immune response including inhibiting the function of CD8^+^ T cells [53], CD4^+^ T cells, NK cells as well as B cells, and promoting CD4^+^ T cells transform into Treg cells may represent potential tumorigenic mechanisms. Therefore, blocking direct cell-to-cell contact and MDSCs recruitment as well as amplification may exhibit an effective treatment strategy of liver cancer.

## Crosstalk of MDSCs with other immune cells

**Fig. 1:**
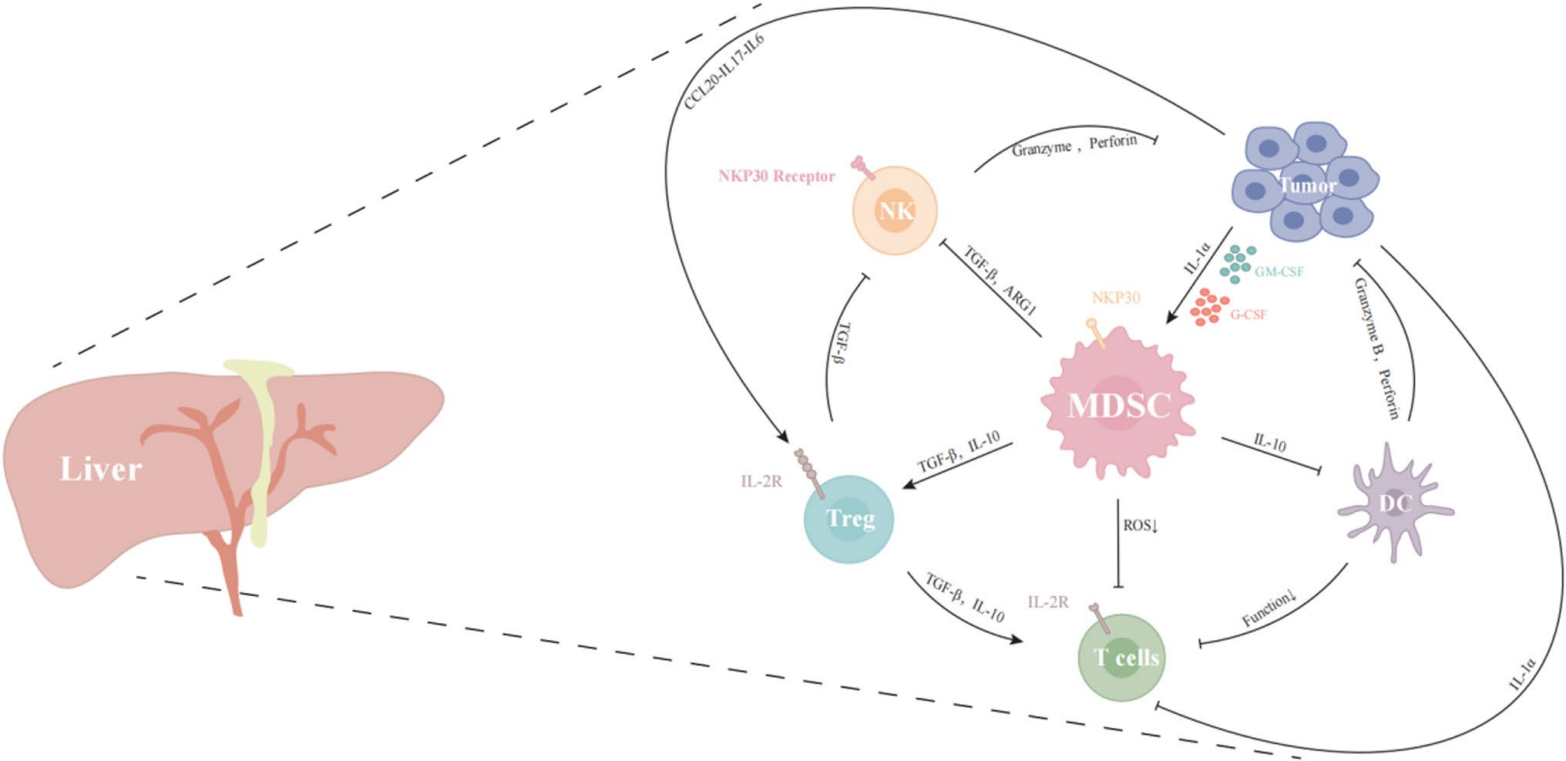
The mechanisms elaborate to MDSC-mediated immunosuppression in Liver cancer. In the TME, GM-CSF and G-CSF released by tumor cells accumulate and recruit MDSCs. MDSCs suppressed the response and proliferation of T and NK cells via TGF-β, Arg-1 or ROS. MDSCs derived IL-10 and TGF-β accelerate accumulation and immunosuppression of Treg. Similarly, MDSCs derived IL-10 inhibit DC function as well. IL1-α promotes MDSCs recruitment and immunosuppression and suppresses T cells function. Tumor-derived CCL20, IL-17 and IL-6 promote Treg expand and immunosuppression. NK cells can lyse tumor cells by direct contact and release of perforin and granzyme B. Treg cells deprivate for IL-2 to T cells and NKP30 on MDSCs binds to receptors on NK cells to suppress NK function. These constitute the immune microenvironment of liver cancer

Comprehensive multi-omics and single-cell examination of liver cancer has confirmed the existence of immunosuppressive cell populations, involving in Treg and MDSCs, which can lead to organismal immune dysfunction. MDSCs have an important influence on the immune modulation of the body, and nowadays targeting therapy MDSCs has become one of the studies hotspots in the field of oncology. Here, we mainly elaborate that MDSCs can block the response of CD4^+^ T cells, CD8^+^ T cells, NK cells as well as DCs directly or indirectly, and improve the proliferation of Treg cells, thus exerting a powerful immunosuppressive function (Fig. 1) [72].

### T cell

Tumor-associated recruitment of MDSCs also suppresses the amount and function of CTL. For the last few years, many research results have confirmed that MDSCs in TME are more immunosuppressive than MDSCs in lymphatic organs and blood. Because it was caused by the low oxygen levels in tumor tissues, which have a regulatory effect on the enhanced inhibitory ability of MDSCs. Suresh Kalathil et al. showed that immunosuppressive cells and cytokines involving in MDSCs, Treg, TGF-β and IL-10 mediated the function of anti-tumor T cells in advanced HCC patients due to the inhibition of cytokine release of cytotoxic T cells. Therefore, depletion of endogenous inhibitory cell populations and restoration of T cell antitumor effect is a therapeutic strategy in liver cancer [73]. In the Akt/N-RAS-induced liver cancer mouse model, myeloid-derived TAMs and MDSCs were found to be co-cultured with T cells, respectively, and the conclusion confirmed that the inhibitory ability of MDSCs was better than that of TAM [74]. In addition, Jianpi Huayu Decoction have been found to promote the differentiation of MDSCs extracted from the spleen of murine hepatoma toward macrophages and DCs, and suppress the generation of ROS. Therefore, the suppression ability of MDSCs to CD4^+^ T cells was weakened [75]. Tumor-released IL-1α accelerates the progression of HCC by promoting MDSCs recruitment and infiltration in spleen and tumor via CXCR2 and suppressing the capacity of CTL [76]. As early as 2008, CD4^+^ T cells co-cultured with MDSCs were confirmed to have increased population of CD25^+^ and FOXP3^+^ cells in clinical liver cancer samples [51]. Immunosuppressive factors secreted by MDSCs, involving in TGF-β and IL-10, both of which promote Treg proliferation. Studies of advance HCC patients have confirmed that increased levels of Treg expression and remarkably improved in the proportion of circulating MDSCs in the blood [73].

### DCs

As the most effective APC, the DCs are recognized to have an important influence on mediating the intrinsic and adaptive immune response. The accumulation of MDSCs recruited into the TME reduces antigen-presentation and migration of DCs, and limits the response to CD8^+^ T and NK cells. DCs dysfunction is unable to present antigen to T cells, resulting in T cell apoptosis and inactivation. It is confirmed that MDSCs suppress the activation, expansion and function of DC by increasing IL-10 level [77]. In addition, the percentage of Treg and MDSCs was remarkably accumulated in AD-mIL12-DC-treated tumors, which increased intratumoral immunosuppression [78].

### NK

NK cells are an important member of a large family of human immunologic effector cells, and activated to secrete cytokines, involving in granzyme B and perforin, which have an important influence on regulating the immunity of the body [79]. Regulatory NK cells (NK_reg_) in the immunosuppressive network can also activate MDSCs and specifically limit the function active of NK cells under different pathological conditions. In mouse models of orthotopic implant HCC, tumor cells associated MDSCs can exert an inhibitory function on NK cells through TGF-β on the membrane [80]. Furthermore, the activity and function of NK cells was found to be suppressed in liver cancer patients from peripheral blood provided by healthy donors and liver cancer patients, and NKp30 on MDSCs was found to inhibit NK cells toxicity and cytokine release by binding to the NKp30 receptor in vitro study [81]. Moreover, clinical data show that about 20% of viral hepatitis progresses to cirrhosis and roughly 10%-20% of these cirrhotic patients may evolute liver cancer. An increasing of research has indicated that MDSCs can suppress NK cell effect, for example, Celeste C Goh et al. showed that HCV can induce CD33^+^CD11b^lo^HLA-DR^lo^ MDSC inhibits mTOR activation via Arg-1 to suppress NK cells release of IFN-γ [82].

## Signaling pathways underlying MDSC-mediated immunosuppression in liver cancer

The amplification and activation of MDSCs are regulated by signaling pathways in the microenvironment, including inflammatory microenvironment and TME. Some studies suggest that inflammatory factors or tumor-derived cytokines can block the further differentiation into mature myeloid cells, resulting in rapid proliferation and differentiation of these cells into MDSCs. Multiple signaling pathways like JAK/STAT, NF-κB and PI3K/Akt are participated in regulating the recruitment and immunosuppressive ability of MDSCs. Regulation of these signaling pathways by small molecules or inhibitors reduces recruitment of MDSCs (Table 1).

**Table 1:** Signaling pathway involved in influence of MDSCs in liver cancer

Signaling pathway	Role	Intervention way	References
AKT/STAT3/ERK	Upregulate in MDSC accumulation and expansion	Gansui-Banxia Decoction	[85]
GM-CSF/JAK2/STAT3	Expression GM-CSF, IDO and PD-L1 in L-MDSC	STAT3 inhibition JAK2 inhibition	[86]
HIF/ENTPD2/CD39L1	Block the differentiation and maintenance of MDSCs	ENTPD2 inhibition	[88]
αKG/HIF1α/CSF-1	Recruit TAMs and MDSCs accumulation	CSF1R inhibitor	[90]
NF-κB	Secretion IL-1α, IL-1β, and IL-6 cytokines and recruitment MDSCs and Treg	–	[93]
5-LO/LTB4/BLT2	Hyperactivated 5-LO metabolism in CD33^+^ MDSCs	–	[99]
TLR4/CXCL1/CXCR2	Accumulation of CXCR2 + PMN-MDSCs	Neomycin	[101]
CXCL10/TLR4/MMP14	Mobilized and recruited M-MDSC	–	[106]
p38 MAPK	Induces M-MDSC accumulation and immunosuppression function	i-BET762 PD-L1	[110]
Mettl1/TGF-β2/PMN-MDSC	Up-regulate PMN-MDSCs and reduced CD8^+^ T cells	Anti-Ly6G antibody, knockdown of Mettl1 or Tgfb2 blockade TGF-β signaling	[109]

### STAT3

The STAT3 pathway have an important impact on regulating the immunosuppressive activity and recruitment of MDSCs [83, 84]. Blockade of STAT3 in tumor-associated myeloid cells increases the turning IMCs to DCs and TAMs, and decreases the recruitment of MDSCs to eliminate immunosuppressive function. According to a recent study, GSBXD (Gansui-Banxia Decoction extraction) exhibited anti-tumor immune activity by blocking AKT/STAT3/ERK signaling pathway, limiting IL-1β and IFN-γ level, and decreasing the accumulation and growth of MDSCs in hepatoma-bearing mice [85]. M Thorn et al. demonstrated that cancer cells secreting GM-SCF activate the JAK2/STAT3 signaling pathway, induce STAT3 phosphorylation in liver MDSCs and translocate to the nucleus to bind to the IDO1 and PD-L1 promoters to perform immunosuppressive functions in mouse liver [86]. Some studies establishing liver metastasis models demonstrated that STAT3 inhibitors increased the level of p38 and JNK, decreased the level of Bcl-2 and upregulated the level of Bax, activated caspases-dependent pathways leading to apoptosis in MDSCs [87]. STAT3 oligonucleotide inhibitor danvarisen (AZD9150) was assessed in clinical tests (NCT01839604) in patients with metastatic HCC [84].

### HIF

Hypoxia-inducible factor α (HIF-1α) is a key factor of MDSCs differentiation and immunosuppressive level in the TME, which promotes the turning M-MDSCs into TAMs through mechanisms associating with CD45 tyrosine phosphatase activity and reduction of STAT3 activity. The CCL26 binds tightly to HIFs and recruits MDSCs to hypoxic regions of the tumor, indicating that hypoxic mechanisms are closely related to tumor infiltration of MDSCs in HCC-bearing mice models [88]. It has been reported that hypoxia-inducing factors have been shown to regulate the expression and differentiation of MDSCs [89]. It has been shown that HIF-1α binds to the promoter Hypoxia responsive elements (HREs) of ENTPD2 to initiate transcription in HCC cells. And ENTPD2 transform extracellular ATP into 5'-AMP, impeding MDSCs from differentiating and maintaining MDSCs immunosuppressive function [88]. He Qin et al. indicated that IL-1β lead to SLC7A11 overexpression of αkg-HIF1α axis upregulated PD-L1 and CSF1, which promoted recruitment of TAM and MDSCs, inducing liver metastasis [90]. The conclusion indicated that hypoxia facilitates the aggregation and infiltration of immunosuppressive MDSCs in tumor cells, thus making it possible for tumor cells to undergo immune escape.

### NF-κB

The NF-κB pathway is involved in various diseases, such as tumors, chronic inflammation, viral infections, and autoimmune diseases, which has been intensively research [91, 92]. The absence of SPTBN1 increases the stability of p65 protein through inhibition of SOCS1 and improves the activation of NF-κB. This process stimulates the level of inflammatory factors in liver, promoting MDSCs and Treg cell accumulation, thus accelerating the formation and progression of HCC [93]. In addition, G9-mediated H3K9 dimethylation (H3K9me2) silences SLC7A2 expression, and SLC7A2 absence mediates CXCL1 upregulation via the PI3K/Akt/NF-κB pathway, recruiting MDSCs and promoting HCC growth and metastasis [94]. It has also been shown that in mouse models of HCC, NF-κB signaling upregulates A3B, which interacts with PRC2 to inhibit H3K27me3 in order to increase CCL2 mRNA and protein levels, thereby recruiting TAMs and MDSCs to tumors [95]. Porta et al. found that tumor-derived PGE2 facilitates p50 NF-kB mediated inhibitory function of MDSCs. Inhibition of the PGE2/p50/NO axis suppresses MDSCs function and restores the anti-tumor effects resulting from immunotherapy [96].

### Metabolic signaling pathways

Tumor cells go through metabolic reprogramming to provide essential capacity and biomolecules for tumor proliferation in order to meet the requirements of their own rapid proliferation. An increasing of evidence showed that dysregulation of energy metabolism in the human body have an important influence on tumorigenesis and survival by generating inhibitory TME that inhibit anti-tumor immune responses [97].

Metabolism of Arg-1 and IDO1 in TME is an important player in MDSC-induced immunosuppression. These two enzymes are strongly expressed in MDSCs and can significantly suppress the proliferation of T cells. L-arginine is a substrate for both arginines, especially Arg-1 and INOS [98]. MDSCs activation promotes L-Arg catabolism and suppresses T and NK cell functions through upregulation of Arg-1. Further, the Arg-1 downregulates the expression of the T cell receptor CD3ε and ζ chain, a key component of the TCR signaling complex, thereby impairing T cell function [138, 139]. MDSCs was revealed to be dependent on IDO1 support and play an important role in forming the immunosuppressive TME. Smith et al. showed that impaired function of circulating MDSCs isolated from Idol^−/−^ mice, which weakens suppression of T cells.

There are several metabolic signaling pathways that affect the immunosuppressive function of MDSCs. In a preclinical in situ ICC patient-derived CAF cells co-injected with tumor QBC939 cells increased cancer stemness, and Gr-1 + MDSCs deletion attenuated this effect. Furthermore, conditioned medium (CM) of CAF educated MDSCs increased the level and activity of 5-LO in MDSCs, thereby enhancing the stemness of MDSCs, and determined that CAF have an important influence on the formation of ICC stemness by mediating the overactivated 5-LO/LTB4-BLT2 metabolism in MDSCs [99]. MDSCs has been reported to suppress tumor-infiltrating lymphocytes by secreting specific metabolites [100]. At present research by Tobias Baumann et al. confirmed that MDSCs present in tumor patients block the metabolic function of T cells by accumulating pyruvate, which exhaust L-arginine or loses L-arginine protein function [15]. New studies have shown that mice with PSC and colitis have decreased intestinal barrier function, resulting in the presence of gut-derived bacteria and lipopolysaccharides (LPS) in the liver, leading to increased accumulation of PMN-MDSC to control hepatocytes to form an immune microenvironment, resulting in hepatocarcinogenesis [101]. Microbiota abnormalities caused by intestinal barrier damage observed in Nlrp6^−/−^ mice recruit the expansion of hepatic M-MDSC and suppress the proliferation and function of T cells, accelerating the progression of HCC [102].

In addition, the environmental sensor General Control Nonderepressible 2 (GCN2) and ferroptosis process are also very important parts of the metabolic signaling pathway affecting the function of MDSCs. Halaby et al. found that GCN2 regulates the immunosuppressive function of TAMs and MDSCs by facilitating the translation of CREB-2/ATF4 transcription factors. However, deletion of GCN2 abolished the suppressive function of MDSCs and increased the infiltration of CD8 + T cells, which exerted anti-tumor function [103]. Further, Kim R et al. reported that ferroptosis is closely associated with the immunosuppressive function of PMN-MDSC in tumors. The genetic and pharmacological inhibition of ferroptosis eliminates the suppression function of PMN-MDC and inhibits the growth of tumors. In addition, it also exerts antitumor effects in combination with PD-1 antibodies [104].

### Pattern recognition receptors

PRRs are a class of receptors expressed primarily on the surface of intrinsic immune cells and are recognized by natural immune cells as DAMPs and PAMPs to initiate immune and inflammatory processes, which consist of five families: TLRs, RLRs, ALRs, NLRs and CLRs [105]. Activation of cell surface TLRs has been reported to contribute more to the suppressive function and tumor-promoting effect of MDSCs. In contrast, TLR activation mainly reduces the ability of MDSCs and exerts anti-tumor effects in vivo. Liu et al. reported that CXCL10 promotes apoptosis of hepatocytes via TLR4 on MDSCs. In rodent models, expression of increased TLR4 promotes tumor recurrence. Inhibition of TLR4 or CXCL10 significantly suppresses the recruitment of M-MDSC and the recurrence of liver tumors after orthotopic liver transplantation [106]. NLRs activation enables MDSCs recruitment and subsequent accumulate to the peritoneal cavity as well as increases Arg-1 expression for immunosuppressive functions, thereby driving tumor progression [107]. RIG-I/MDA5 antagonists reduce secretion of chemokines to limit MDSCs recruitment, thereby increasing the efficacy of CAR-T cells [105, 108].

### Other potential

In addition to the above studied mechanisms, a number of preclinical research have explored that MDSCs also promote tumor development of HCC via multiple signaling pathway, involving in p38 MAPK, TGF-β, etc. It was found that insufficient Radiofrequency ablation (iRFA) treatment exhibited liver tumor proliferation and metastasis, recruited PMN-MDSC expansion, dampened CD8^+^ T cells response, and blockade of the Mettl1/TGF-β2/PMN-MDSC axis alleviates tumor development [109]. In two models of liver fibrosis, hepatic stellate cells (HSC) induce expansion and immunosuppression of M-MDSCs through p38 MAPK-mediated enhancer reprogramming. Treatment with p38 MAPK inhibitors can eliminate the interference of HSC M-MDSC and thus inhibits the growth of HCC [110]. The level of MDSCs was remarkably increased of recurrent liver transplantation patients, which may have an important influence on the regulation of TLR4 by CXCL10, a finding that was further validated in rat models of liver transplantation. Liu et al. showed that CXCL10/TLR4/MMP14 signaling pathway recruit M-MDSCs was responsible for the recurrence of hepar metastases in 331 HCC patients [106]. Previous studies have confirmed that FGL2 can regulate the tumor immune microenvironment to accelerate cancer development. In the orthotopic liver cancer mouse model, FGL2 maintains the undifferentiated state of BM cells to promote the accumulation of MDSCs and thus the development of HCC [111]. There are also a number of non-traditional signaling pathways including apoptosis proteins regulate MDSCs function. According to Haverkamp, death signaling pathways are closely associated with the growth, survival and function of MDSCs. c-FLIP and Mcl-1 are apoptosis inhibitory proteins that regulate tumor apoptosis and necrosis signaling pathways, which affect the suppressive function of MDSCs [112]. Fiore et al. reported that FLIP expression in M-MDSC can confer immunosuppressive properties to suppress T cell proliferation, and targeting FLIP therapy can eliminate the immunosuppressive function of M-MDSC. In addition, the combination of inhibitors of FLIP with immune checkpoints also increases the immunotherapy [113].

In conclusion, the expression and mechanism of STAT3, HIF, NF-κB, metabolic pathways, pattern recognition receptors, etc. will recruit MDSCs, increase the expression levels of Arg1, INOS, PD-L1, make them accumulate and expand rapidly in the tumor microenvironment, increase their immunosuppressive function as well as inhibit the role of CD8^ +^ T, thus promote tumor progression.

## Basic strategies to therapeutically target MDSCs

**Fig. 2:**
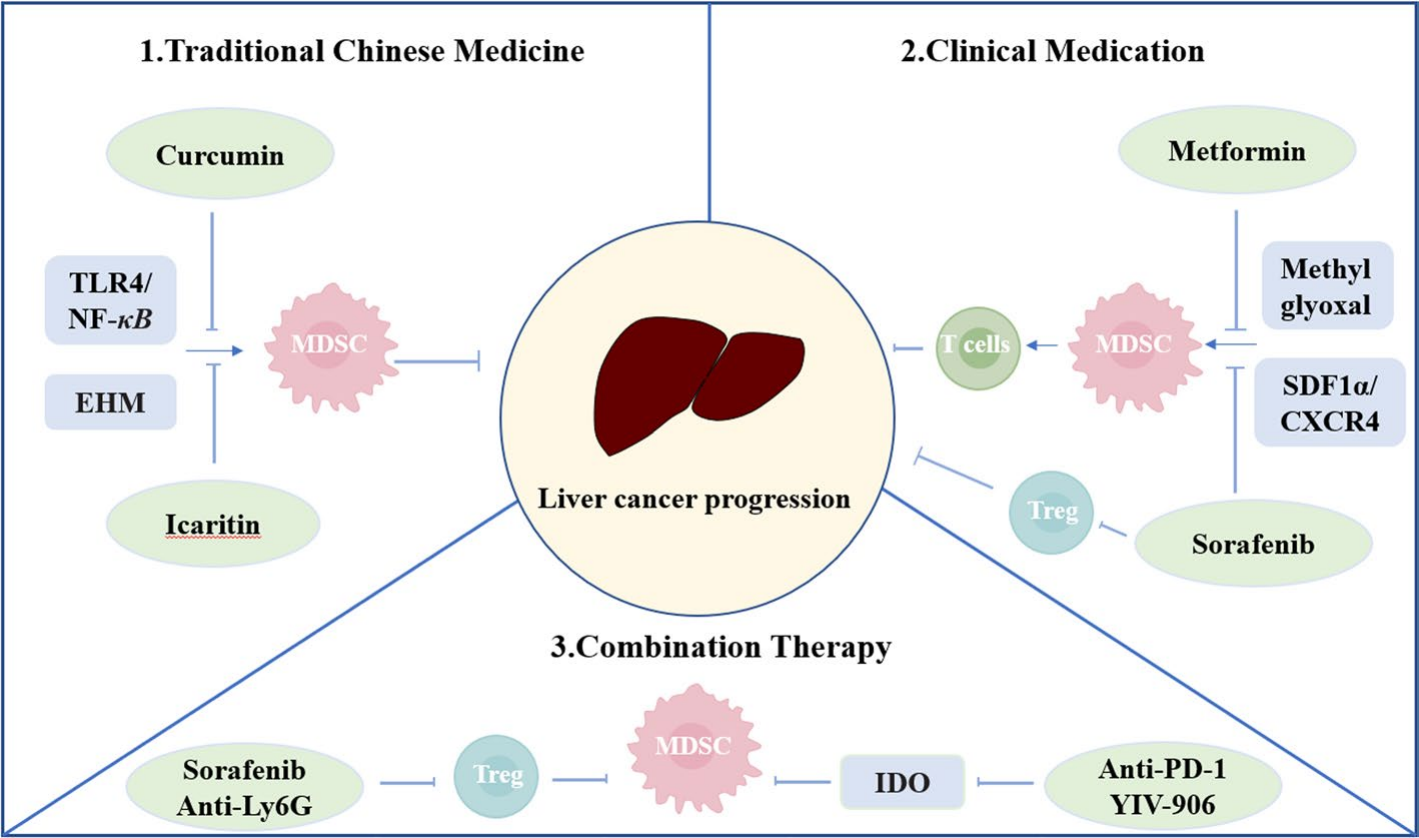
Basic therapeutic strategy of MDSCs could be approximately classified into three parts: Traditional Chinese Medicine; Clinical medication; Combination Therapy. EMH: extramedullary hematopoiesis; IDO: indoleamine 2,3-dioxygenase;

MDSCs are a heterogeneous population of cells from the BM that suppressed the functions of immune effector cells [114]. The enrichment and activation of MDSCs take part in different regulating immune effector cells and malignant cells. MDSCs has strong immunosuppressive activity, supports immune escape, and promotes tumor metastasis as well as angiogenesis through various non-immune activities. Therefore, inhibition of MDSCs is also one of the effective ways to combat tumors [23] (Fig. 2, Table 2).

**Table 2:** Therapeutic strategy of targeting MDSCs in solid tumors

Preclinical models	Therapeutic strategy	Role	References
HepG2 subcutaneous mouse liver cancer models	Curcumin	Suppressed MDSCs expansion and the release of correlated factors GM-CSF and G-CSF	[119]
Hepa1–6 cells orthotopic mouse liver cancer models	Icaritin	Reduced the accumulation and activation of MDSCs, and improved the accumulation and response of CTL	[120]
Samples from liver cancer patients	Metformin	Reversed the effect of MDSCs to T cells	[123]
Ncoa5^+/−^ Male Mice orthotopic mouse liver cancer models	Metformin	Reduced intrahepatic MDSCs and M2 macrophages	[127]
BNL 1ME A.7R.1 cells subcutaneous mouse liver cancer models	Sorafenib	Decreased Tregs and MDSCs	[39]
BNL cells orthotopic mouse liver cancer models	anti-Ly6G Sorafenib	Down-regulation the cell population of Ly6G + MDSCs and enhanced the T cells proliferation	[131]
Hepa 1–6 subcutaneous mouse liver cancer models	Sorafenib compound kushen injection	Diminished MDSCs	[132]
Hepa 1–6 subcutaneous mouse liver cancer models	anti-PD1 YIV-906	Reducing monocytic MDSC, M2- macrophage and M1-like macrophages	[134]

### Traditional Chinese medicine therapy

In recent years, the exploring natural products to enhance immunity has become a worldwide research hotspot [115]. The Traditional Chinese medicines (TCMs) have remarkable features such as low drug resistance, low toxic side effects and long-lasting effects.

Data show that some TCMs has the function of killing and inhibiting the proliferation of tumor cells, among which improving the immune microenvironment to accelerate immune effector cells to kill tumor cells is a significant research direction at present [116]. Some herbal medicines (alkaloids, polysaccharides, polyphenols) have been reported to improve the humoral immune functions of the body, such as improving the functions of CTL, NK cells, DCs, TAMs as well as MDSCs [117].

In preclinical tumor models, curcumin and Icaritin reduced tumor immunosuppressive mechanisms by inhibiting MDSCs function. Curcumin [118] was shown to dampen the growth of hepatic tumor cells via regulating the TLR4/NF-κB signaling mechanisms in a xenograft liver cancer model, possibly by reducing the releasing of related factors such as GM-CSF and G-CSF, reducing recruitment and mobilization to MDSCs, and reducing the immunosuppressive effect of MDSCs [119]. The Icaritin is an efficacious component obtained from the TCMs Epimedium by intelligent Chinese scientists and has been approved for marketing. A study demonstrated that in tumor-bearing mice in situ and transplanted liver cancer models, Icaritin dampened tumor-associated splenic EMH significantly reduced MDSCs accumulation in tumors and spleens, and improved the amount and function of CTL, thereby inhibiting HCC development and prolonging the survival of mice [120].

### Clinical medication

#### Metformin

Metformin, an oral medication for the treatment of diabetes, has been in clinical application for more than 50 years. It has been showed significantly that metformin can have beneficial effect on anti-tumor by enhancing immunity [51]. Some reports have shown that metformin can exert antitumor role in mice with normal immunity, but does not play an obvious antitumor role in mice with immune deficiency, indicating that metformin is supported by antitumor effects through immunity [121, 122]. Recent studies have shown that metformin can restore the targeted killing ability of CTL by reversing the inhibitory function of M-MDSC of tumor tissue origin in patients by neutralizing pyruvate aldehyde when studying samples from patients with HCC [123]. Chronic hepatitis can promote the development of HCC through several mechanisms, one of the reasons being the development of an immunosuppressive environment, for example, leading to an increase in MDSCs that evade immune surveillance [124, 125]. Metformin prophylaxis ameliorates the chronic inflammatory response of pre-cancerous intrahepatic immune cells, a finding that was demonstrated in vivo experiments. An increasing proportion of MDSCs was found in Ncoa5 ± male mice of liver, and metformin reversed the result. Metformin remarkably downregulating the population of myeloid cells and MDSCs in the liver to prevent the development of HCC [126].

#### Sorafenib

Multikinase inhibitor sorafenib has demonstrated hopeful results in the therapy of patients with HCC, significantly extending their survival. It is a first-line clinical therapy option for HCC [127]. Previous studies reported that sorafenib can exert anti-tumor effects in the aspect of both inhibition of tumor cell growth and angiogenesis. It can also regulate various of effector cells to activate the immune system. Mengde Cao et al. confirmed that sorafenib can reduce the amount of MDSCs and Treg cells in mouse HCC models, and these two kind immune cell populations were positively correlated with tumor size [39]. In two major clinical trials (SHARP and phase III of AP), sorafenib frequently developed resistance during treatment, which may be related to CD11b^+^Gr1^+^ myeloid cell infiltration into tumor tissues [128]. Yunching Chen et al. found that sorafenib treatment increased Gr-1 + myeloid cell infiltration in orthotopic HCC mouse models, thereby expressed higher levels of multiple pro-fibrotic factors, including SDF1α and CXCR4. The Gr-1 + MDSCs mediate hepatic tumor resistance to sorafenib by increasing hepatic stellate cell survival, differentiation, and induction of tumor fibrosis. Targeting the SDF1α/CXCR4 pathway or blocking Gr-1 + MDSCs infiltration might be an effective way to overcome resistance to sorafenib therapy [129].

#### Combination therapy

Many studies have shown that, long-term administration results in drug resistance for most chemotherapeutics, probably due to treatment leading to BM suppression and altered immune cell function. However, combination of drugs significantly improves the therapeutic effect of chemotherapeutic agents, enhances tumor suppression, reduces toxic side effects, and reduces BM suppression, etc. Chun-Jung Chang et al. demonstrated that Ly6G + MDSCs with tumor-infiltrating were improved in sorafenib treated liver tumors in situ, and that these cells with immunosuppressive properties attenuated the therapeutic effect of sorafenib. Nevertheless, Sorafenib coupled with Anti-Ly6G or Anti-IL-6 can improve the treatment of Ly6G + MDSC in orthotopic HCC mice [130]. In particular reduction of MDSCs in some HCC patients treated with sorafenib may have been measured early and increased in MDSCs when acquired at a later time point, explaining the different effects obtained with the same chemotherapeutic agent. Increasingly, studies have shown that the combination of TCMs and chemotherapeutics can reduce the dose of chemotherapeutics administered, improve adverse effects and patients' quality of life, and increase the antitumor effect. Yangyang et al. confirmed that the combination of compound kushen injection and sorafenib reshaped the TME, increased the anti-tumor abilities of sorafenib, and reduced adverse reaction, blocking the recruitment of MDSCs [131]. IDO, an immune checkpoint protein, provides immunosuppression through the mechanism of recruitment and activation of Treg and MDSCs [132]. It was showed that combination of anti-PD-1 and YIV-906 administration significantly reduced IDO and M-MDSC of Hepa 1–6 tumor, enhanced IFNg action, polarized macrophages toward M1 type, and enhanced anti-tumor effects [133].

#### Innovative technologies therapy

We currently lack tools to provide an elaborated description of the patterns of MDSCs activation and immunosuppression. However, the use of some new techniques such as scRNA-seq, spatial transcriptomics and multi-parametric immunohistochemical analysis can be very enlightening in terms of the quantitative tools that can be applied to identify the type of inhibition patterns. Darden et al. performed a preliminary study using single-cell RNAseq to establish the transcriptomic landscape of MDSCs subpopulations in sepsis [134]. In addition, single-cell sequencing was used to form a comprehensive single-cell landscape to determine the relationships between lymphocytes and myeloid cells after liver transplant rejection [135]. It was showed that PDGF stimulation promoted chemokines release increased recruitment to PMN-MDSC, and results using spatial transcriptome and single-cell sequencing showed that inhibition of PDGFRa/b reduced the suppression of the immune microenvironment [136].

Moreover, Arg-1 is highly expressed in MDSCs, and the enzyme depletes L-arginine, which can lead to T-cell suppression. Consequently, based on immunization tools, vaccination, antibody or nanoparticle-targeted Arg-1 therapies may also be potential emerging approaches for the treatment of MDSCs. Aaboe Jørgensen M. et al. reported that immunomodulatory capacity of myeloid cells isolated from vaccinated mice was significantly impaired and exerted an anti-tumor effect [137]. Canè S. et al. reported a new pathway for generating an active form of hArg1 whose negative impact on modulating immunity can be targeted by hArg1 monoclonal antibodies, and this can be combined with immunotherapy as an anti-tumor strategy [138]. Besides, nanoparticle-targeted MDSCs approaches also deserve to be investigated. The exploration of nanoparticle systems for effective imaging, localization and treatment of MDSCs will represent a strategic therapeutic innovation to improve tumor treatment [139]. For example, LIC has been reported to exert anti-tumor effects by targeting PI3Kγ-AKT signaling pathway, reducing the expression of Arg1 and ROS, promoting apoptosis of MDSCs cells, and reducing the inhibition of CD8 + T cells [140].

## Discussion and future perspectives

Liver cancer is an extremely high malignant tumor disease with the incidence rate among cancers and the mortality rate has been increasing all over the world. Due to the hidden nature of liver cancer, it is usually detected at an advanced stage with poor prognosis and lack of effective treatment. More and more studies have shown that immunotherapy has become the one of first-line treatments for liver cancer, but the effectiveness of treatment is still limited. Thus, improving immune system of patients will respond well to therapy with advanced liver cancer.

MDSCs are heterogeneous immature cells and an indispensable element of the TME, which are involved in bad prognosis in HCC patients. Currently, suppression of MDSCs expansion, function, metabolism, and promotion of MDSCs differentiation and apoptosis are the basic measures for the therapy for liver cancer. Nevertheless, MDSCs have different subtypes and are phenotypically similar to normal myeloid cells and neutrophils, making it more difficult to target MDSCs. In clinical trials, neutrophils and PMN-MDSC can be distinguished by gradient centrifugation. But this method also had some disadvantages. Because there will be activated neutrophils in the low-density area, which will lead to miscalculation of the cell ratio. In addition, variations in the PMN-MDSC ratio can also be caused by storage conditions and sample handling methods. Similarly, we should also consider the impact of liver tumor site and stage, pathology type, and drug treatment on MDSCs in clinical trial studies [26, 61, 141, 142]. At present, most studies focus on regulating MDSCs as a whole; however, the mechanisms regulated by different MDSCs subtypes may differ. Only a few studies have explored MDSCs with tumor infiltration, probably own to the difficulty and exorbitant price of isolating MDSCs, and their complex attachment to tumor cells as well as their small proportion, thus effects and inhibitory mechanisms of MDSCs need to be elaborated in detail [26, 143, 144].

MDSCs and other immune cells form a complex TME. This paper reviews the crosstalk between MDSCs and CD8^+^T, CD4^+^T, Treg, NK and DCs in liver cancer and shows how they have influence on the development of liver cancer. Increasing the amount and function of MDSCs decreases the response of CTL because cysteine required for T cell activation has been deprived. Preclinical studies suggest that increased Treg may be associated with MDSCs-induced production of Foxp3^+^ Treg via IL-10 and TGF-β, MDSCs also amplify the number of Treg already present in vivo. Increased MDSCs enhance competition between MDSCs and DCs and inhibit DCs maturation and antigen-presenting functions. Therefore, MDSCs are inseparable from T cells, NK cells, as well as DCs, and the quantity and function of MDSCs influence the multiplication and efficacy to other effector cells. The specific mechanism for how MDSCs regulated DCs and Treg cells remains unclear in liver cancer.

Various chemotherapeutic drugs, checkpoint Inhibitors, herbal medicines, and small molecule compounds are currently demonstrated to suppress the proliferation and immunosuppressive level of MDSCs in liver cancer. Single drug therapy seems to be unable to achieve ideal effect for MDSCs treatment with complex mechanism, such as clinical drugs for liver cancer, including sorafenib, lenvatinib, PD-1 inhibitors, and curcumin, etc. The former may produce drug resistance and some toxic side effects. And Chinese herbal medicine although with less toxic side effects, does not work as fast as chemotherapeutic drugs. Current research shows that the respective mechanisms of combination drugs produce synergistic effects, reduce toxic side effects and improve therapeutic effect. However, not all combination drugs can produce synergistic effects. It has been reported that 5-FU induces the increasing of MDSCs and impairs antitumor efficacy of PD-L1 blockers in HCC [91, 144]. It is worth noting that there is not a clear indication of the dose and ratio of combination drugs, so the drug dose dosage, timing of administration and treatment sequence should be carefully determined when combining drugs.

With the development and innovation of research in the field of oncology, there will be more and more drugs targeting MDSCs. For the advancement of the better clinical results, it is indispensible to elucidate the mechanism of MDSCs in the microenvironment and develop as well as optimize the existing therapeutic regimens so as to serve the oncology patients better and maximize their lives.

## Data Availability

Data sharing not applicable to this article as no datasets were generated or analyzed during the current study.

## Abbreviations

MDSCs: Myeloid-derived suppressor cells
DCs: Dendritic cell
HCC: Hepatocellular carcinoma
ICC: Intrahepatic cholangiocarcinoma
Treg: Regulatory T cells
TAMs: Tumor-associated macrophages
TME: Tumor microenvironment
LSEC: Liver sinusoidal endothelial cells
KC: Kupffer cells
NK: Nature kill
NKT: Nature kill T
PD-1: Programmed death-1
CTLA-4: CTL-associated antigen-4
Arg-1: Arginase-1
INOS: Inducible nitric oxide synthase
NO: Nitric oxide
ROS: Reactive oxygen species
HSCs: Hematopoietic stem cells
LOX-1: Lectin-type oxidized LDL receptor 1
TGF-β: Transforming growth factor
IL-10: Interleukin 10
FGL2: Fibrinogen-like protein 2
GM-CSF: Granulocyte macrophage colony stimulating factor
G-CSF: Granulocyte colony stimulating factor
EMH: Extramedullary hematopoiesis
IDO: Indoleamine 2,3-dioxyenase
TCR: T cell receptor
APC: Antigen-presenting cells
CTL: Cytotoxic T-Lymphocyte
BM: Bone marrow
TLRs: Toll-like receptors
NLRs: Nucleotide oligomerization domain (NOD)-like receptors
RLRs: Retinoic acid-inducible gene-I (RIG-I)-like receptors
CLRs: C-type lectin receptors
ALRs: Absent in melanoma-2 (AIM2)-like receptors

## References

[1] Li X, Ramadori P, Pfister D, Seehawer M, Zender L, Heikenwalder M (2021). The immunological and metabolic landscape in primary and metastatic liver cancer. Nat Rev Cancer.

[2] Mintz KJ (1876). Leblanc RM (2021) The use of nanotechnology to combat liver cancer: progress and perspectives. Biochim Biophys Acta Rev Cancer.

[3] Cioarca-Nedelcu R, Atanasiu V, Stoian I (2021). Alcoholic liver disease-from steatosis to cirrhosis—a biochemistry approach. J Med Life.

[4] Lin XM, Hu L, Gu J, Wang RY, Li L, Tang J (2017). Choline kinase α mediates interactions between the epidermal growth factor receptor and mechanistic target of rapamycin complex 2 in hepatocellular carcinoma cells to promote drug resistance and xenograft tumor progression. Gastroenterology.

[5] Llovet JM, Kelley RK, Villanueva A, Singal AG, Pikarsky E, Roayaie S (2021). Hepatocellular carcinoma. Nat Rev Dis Primers.

[6] Xie Y, Zhang L, Li YY, He D, Zheng LF (2021). Chrysophanol localizes in mitochondria to promote cell death through upregulation of mitochondrial cyclophilin D in HepG2 cells. Chin Herb Med.

[7] Feng J, Li J, Wu L, Yu Q, Ji J, Wu J (2020). Emerging roles and the regulation of aerobic glycolysis in hepatocellular carcinoma. J Exp Clin Cancer Res.

[8] Chen W, Chiang CL, Dawson LA (2021). Efficacy and safety of radiotherapy for primary liver cancer. Chin Clin Oncol.

[9] Harris PS, Hansen RM, Gray ME, Massoud OI, McGuire BM, Shoreibah MG (2019). Hepatocellular carcinoma surveillance: an evidence-based approach. World J Gastroenterol.

[10] Schizas D, Mastoraki A, Routsi E, Papapanou M, Tsapralis D, Vassiliu P (2020). Combined hepatocellular-cholangiocarcinoma: an update on epidemiology, classification, diagnosis and management. Hepatobiliary Pancreat Dis Int.

[11] Bruix J, Han KH, Gores G, Llovet JM, Mazzaferro V (2015). Liver cancer: approaching a personalized care. J Hepatol.

[12] Siegel RL, Miller KD, Fuchs HE, Jemal A (2022). Cancer statistics, 2022. CA Cancer J Clin.

[13] Alvarez M, Benhammou JN, Darci-Maher N, French SW, Han SB, Sinsheimer JS, Agopian VG (2022). Human liver single nucleus and single cell RNA sequencing identify a hepatocellular carcinoma-associated cell-type affecting survival. Genome Med.

[14] Qi X, Yang M, Ma L, Sauer M, Avella D, Kaifi JT (2020). Synergizing sunitinib and radiofrequency ablation to treat hepatocellular cancer by triggering the antitumor immune response. J Immunother Cancer.

[15] Sullivan KM, Jiang X, Guha P, Lausted C, Carter JA, Hsu C (2023). Blockade of interleukin 10 potentiates antitumour immune function in human colorectal cancer liver metastases. Gut.

[16] Dika EI, Khalil DN, Abou-Alfa GK (2019). Immune checkpoint inhibitors for hepatocellular carcinoma. Cancer.

[17] Bai Y, Chen D, Cheng C, Li Z, Chi H, Zhang Y (2022). Immunosuppressive landscape in hepatocellular carcinoma revealed by single-cell sequencing. Front Immunol.

[18] Jhunjhunwala S, Hammer C, Delamarre L (2021). Antigen presentation in cancer: insights into tumour immunogenicity and immune evasion. Nat Rev Cancer.

[19] Zajkowska M, Mroczko B (2022). Chemokines in primary liver cancer. Int J Mol Sci.

[20] Greten TF, Lai CW, Li G, Staveley-O'Carroll KF (2019). Targeted and immune-based therapies for hepatocellular carcinoma. Gastroenterology.

[21] Zhai J, Song Z, Chang H, Wang Y, Han N, Liu Z (2021). He-Wei Granule enhances anti-tumor activity of cyclophosphamide by changing tumor microenvironment. Chin Herb Med.

[22] Jia L, Gao Y, He Y, Hooper JD, Yang P (2020). HBV induced hepatocellular carcinoma and related potential immunotherapy. Pharmacol Res.

[23] De Cicco P, Ercolano G, Ianaro A (2020). The new era of cancer immunotherapy: targeting myeloid-derived suppressor cells to overcome immune evasion. Front Immunol.

[24] Mantovani A, Barajon I, Garlanda C (2018). IL-1 and IL-1 regulatory pathways in cancer progression and therapy. Immunol Rev.

[25] Lu X, Horner JW, Paul E, Shang X, Troncoso P, Deng P (2017). Effective combinatorial immunotherapy for castration-resistant prostate cancer. Nature.

[26] Li K, Shi H, Zhang B, Ou X, Ma Q, Chen Y (2021). Myeloid-derived suppressor cells as immunosuppressive regulators and therapeutic targets in cancer. Signal Transduct Target Ther.

[27] Murdoch C, Muthana M, Coffelt SB, Lewis CE (2008). The role of myeloid cells in the promotion of tumour angiogenesis. Nat Rev Cancer.

[28] Tcyganov EN, Hanabuchi S, Hashimoto A, Campbell D, Kar G, Slidel TW (2021). Distinct mechanisms govern populations of myeloid-derived suppressor cells in chronic viral infection and cancer. J Clin Invest.

[29] Nakamura K, Smyth MJ (2020). Myeloid immunosuppression and immune checkpoints in the tumor microenvironment. Cell Mol Immunol.

[30] Rui L (2014). Energy metabolism in the liver. Compr Physiol.

[31] Kubes P, Jenne C (2018). Immune responses in the liver. Annu Rev Immunol.

[32] Trefts E, Gannon M, Wasserman DH (2017). The liver. Curr Biol.

[33] Bogdanos DP, Gao B, Gershwin ME (2013). Liver immunology. Compr Physiol.

[34] Robinson MW, Harmon C, O'Farrelly C (2016). Liver immunology and its role in inflammation and homeostasis. Cell Mol Immunol.

[35] Shaked A, DesMarais MR, Kopetskie H, Feng S, Punch JD, Levitsky J (2019). Outcomes of immunosuppression minimization and withdrawal early after liver transplantation. Am J Transplant.

[36] Oura K, Morishita A, Tani J, Masaki T (2021). Tumor Immune microenvironment and immunosuppressive therapy in hepatocellular carcinoma: a review. Int J Mol Sci.

[37] Thomson AW, Knolle PA (2010). Antigen-presenting cell function in the tolerogenic liver environment. Nat Rev Immunol.

[38] Crispe IN (2011). Liver antigen-presenting cells. J Hepatol.

[39] Cao M, Xu Y, Youn JI, Cabrera R, Zhang X, Gabrilovich D (2011). Kinase inhibitor Sorafenib modulates immunosuppressive cell populations in a murine liver cancer model. Lab Invest.

[40] Zou W (2006). Regulatory T cells, tumour immunity and immunotherapy. Nat Rev Immunol.

[41] González-Navajas JM, Fan DD, Yang S, Yang FM, Lozano-Ruiz B, Shen L (2021). The impact of tregs on the anticancer immunity and the efficacy of immune checkpoint inhibitor therapies. Front Immunol.

[42] Tanaka A, Sakaguchi S (2017). Regulatory T cells in cancer immunotherapy. Cell Res.

[43] Li WM, Liu HR (2016). CCL20-CCR6 cytokine network facilitate Treg activity in advanced grades and metastatic variants of hepatocellular carcinoma. Scand J Immunol.

[44] Ruf B, Heinrich B, Greten TF (2021). Immunobiology and immunotherapy of HCC: spotlight on innate and innate-like immune cells. Cell Mol Immunol.

[45] Wu SY, Fu T, Jiang YZ, Shao ZM (2020). Natural killer cells in cancer biology and therapy. Mol Cancer.

[46] Chen Y, Lu D, Churov A, Fu R (2020). Research progress on NK cell receptors and their signaling pathways. Mediators Inflamm.

[47] Zheng X, Qian Y, Fu B, Jiao D, Jiang Y, Chen P (2019). Mitochondrial fragmentation limits NK cell-based tumor immunosurveillance. Nat Immunol.

[48] Nakamoto N, Cho H, Shaked A, Olthoff K, Valiga ME, Kaminski M (2009). Synergistic reversal of intrahepatic HCV-specific CD8 T cell exhaustion by combined PD-1/CTLA-4 blockade. PLoS Pathog.

[49] Zhou SL, Zhou ZJ, Hu ZQ, Huang XW, Wang Z, Chen EB (2016). Tumor-associated neutrophils recruit macrophages and T-regulatory cells to promote progression of hepatocellular carcinoma and resistance to sorafenib. Gastroenterology.

[50] Fridlender ZG, Sun J, Kim S, Kapoor V, Cheng G, Ling L (2009). Polarization of tumor-associated neutrophil phenotype by TGF-beta: "N1" versus "N2" TAN. Cancer Cell.

[51] Hoechst B, Ormandy LA, Ballmaier M, Lehner F, Krüger C, Manns MP (2008). A new population of myeloid-derived suppressor cells in hepatocellular carcinoma patients induces CD4(+)CD25(+)Foxp3(+) T cells. Gastroenterology.

[52] Shime H, Maruyama A, Yoshida S, Takeda Y, Matsumoto M, Seya T (2017). Toll-like receptor 2 ligand and interferon-γ suppress anti-tumor T cell responses by enhancing the immunosuppressive activity of monocytic myeloid-derived suppressor cells. Oncoimmunology..

[53] Ma T, Renz BW, Ilmer M, Koch D, Yang Y, Werner J (2022). Myeloid-derived suppressor cells in solid tumors. Cells.

[54] Wu Y, Yi M, Niu M, Mei Q, Wu K (2022). Myeloid-derived suppressor cells: an emerging target for anticancer immunotherapy. Mol Cancer.

[55] Ling Z, Yang C, Tan J, Dou C, Chen Y (2021). Beyond immunosuppressive effects: dual roles of myeloid-derived suppressor cells in bone-related diseases. Cell Mol Life Sci.

[56] Barry ST, Gabrilovich DI, Sansom OJ, Campbell AD, Morton JP (2023). Therapeutic targeting of tumour myeloid cells. Nat Rev Cancer.

[57] Dolcetti L, Peranzoni E, Ugel S, Marigo I, Fernandez Gomez A, Mesa C (2010). Hierarchy of immunosuppressive strength among myeloid-derived suppressor cell subsets is determined by GM-CSF. Eur J Immunol.

[58] Kalathil SG, Thanavala Y (2021). Importance of myeloid derived suppressor cells in cancer from a biomarker perspective. Cell Immunol.

[59] Veglia F, Perego M, Gabrilovich D (2018). Myeloid-derived suppressor cells coming of age. Nat Immunol.

[60] Condamine T, Dominguez GA, Youn JI, Kossenkov AV, Mony S, Alicea-Torres K (2016). Lectin-type oxidized LDL receptor-1 distinguishes population of human polymorphonuclear myeloid-derived suppressor cells in cancer patients. Sci Immunol..

[61] Tcyganov E, Mastio J, Chen E, Gabrilovich DI (2018). Plasticity of myeloid-derived suppressor cells in cancer. Curr Opin Immunol.

[62] Krishnamoorthy M, Gerhardt L, Maleki VS (2021). Immunosuppressive effects of myeloid-derived suppressor cells in cancer and immunotherapy. Cells.

[63] Bronte V, Brandau S, Chen SH, Colombo MP, Frey AB, Greten TF (2016). Recommendations for myeloid-derived suppressor cell nomenclature and characterization standards. Nat Commun.

[64] Mabuchi S, Sasano T, Komura N (2021). Targeting myeloid-derived suppressor cells in ovarian cancer. Cells.

[65] Tengesdal IW, Dinarello A, Powers NE, Burchill MA, Joosten LAB, Marchetti C (2021). Tumor NLRP3-derived IL-1β drives the IL-6/STAT3 axis resulting in sustained MDSC-mediated immunosuppression. Front Immunol.

[66] Veglia F, Sanseviero E, Gabrilovich DI (2021). Myeloid-derived suppressor cells in the era of increasing myeloid cell diversity. Nat Rev Immunol.

[67] van Vlerken-Ysla L, Tyurina YY, Kagan VE, Gabrilovich DI (2023). Functional states of myeloid cells in cancer. Cancer Cell.

[68] Ghonim MA, Ibba SV, Tarhuni AF, Errami Y, Luu HH, Dean MJ (2021). Targeting PARP-1 with metronomic therapy modulates MDSC suppressive function and enhances anti-PD-1 immunotherapy in colon cancer. J Immunother Cancer.

[69] Molon B, Ugel S, Del Pozzo F, Soldani C, Zilio S, Avella D (2011). Chemokine nitration prevents intratumoral infiltration of antigen-specific T cells. J Exp Med.

[70] De Sanctis F, Lamolinara A, Boschi F, Musiu C, Caligola S, Trovato R (2022). Interrupting the nitrosative stress fuels tumor-specific cytotoxic T lymphocytes in pancreatic cancer. J Immunother Cancer.

[71] Marigo I, Bosio E, Solito S, Mesa C, Fernandez A, Dolcetti L (2010). Tumor-induced tolerance and immune suppression depend on the C/EBPbeta transcription factor. Immunity.

[72] Yu SJ, Ma C, Heinrich B, Brown ZJ, Sandhu M, Zhang Q (2019). Targeting the crosstalk between cytokine-induced killer cells and myeloid-derived suppressor cells in hepatocellular carcinoma. J Hepatol.

[73] Kalathil S, Lugade AA, Miller A, Iyer R, Thanavala Y (2013). Higher frequencies of GARP(+)CTLA-4(+)Foxp3(+) T regulatory cells and myeloid-derived suppressor cells in hepatocellular carcinoma patients are associated with impaired T-cell functionality. Cancer Res.

[74] Liu YT, Tseng TC, Soong RS, Peng CY, Cheng YH, Huang SF (2018). A novel spontaneous hepatocellular carcinoma mouse model for studying T-cell exhaustion in the tumor microenvironment. J Immunother Cancer.

[75] Xie Y, Zhang Y, Wei X, Zhou C, Huang Y, Zhu X (2020). Jianpi Huayu decoction attenuates the immunosuppressive status of H22 hepatocellular carcinoma-bearing mice: by targeting myeloid-derived suppressor cells. Front Pharmacol.

[76] Lin D, Mei Y, Lei L, Binte Hanafi Z, Jin Z, Liu Y (2022). Immune suppressive function of IL-1α release in the tumor microenvironment regulated by calpain 1. Oncoimmunology.

[77] Hu CE, Gan J, Zhang RD, Cheng YR, Huang GJ (2011). Up-regulated myeloid-derived suppressor cell contributes to hepatocellular carcinoma development by impairing dendritic cell function. Scand J Gastroenterol.

[78] Vogt A, Sievers E, Lukacs-Kornek V, Decker G, Raskopf E, Meumann N (2014). Improving immunotherapy of hepatocellular carcinoma (HCC) using dendritic cells (DC) engineered to express IL-12 in vivo. Liver Int.

[79] Liu S, Galat V, Galat Y, Lee YKA, Wainwright D, Wu J (2021). NK cell-based cancer immunotherapy: from basic biology to clinical development. J Hematol Oncol.

[80] Li H, Han Y, Guo Q, Zhang M, Cao X (2009). Cancer-expanded myeloid-derived suppressor cells induce anergy of NK cells through membrane-bound TGF-beta 1. J Immunol.

[81] Hoechst B, Voigtlaender T, Ormandy L, Gamrekelashvili J, Zhao F, Wedemeyer H (2009). Myeloid derived suppressor cells inhibit natural killer cells in patients with hepatocellular carcinoma via the NKp30 receptor. Hepatology.

[82] Goh CC, Roggerson KM, Lee HC, Golden-Mason L, Rosen HR, Hahn YS (2016). Hepatitis C virus-induced myeloid-derived suppressor cells suppress NK cell IFN-γ production by altering cellular metabolism via arginase-1. J Immunol.

[83] Ma C, Zhang Q, Greten TF (2021). MDSCs in liver cancer: a critical tumor-promoting player and a potential therapeutic target. Cell Immunol.

[84] Lu LC, Chang CJ, Hsu CH (2019). Targeting myeloid-derived suppressor cells in the treatment of hepatocellular carcinoma: current state and future perspectives. J Hepatocell Carcinoma.

[85] Feng XY, Chen BC, Li JC, Li JM, Li HM (2021). Gansui-Banxia decoction extraction inhibits MDSCs accumulation via AKT /STAT3/ERK signaling pathways to regulate antitumor immunity in C57bl/6 mice. Phytomedicine.

[86] Thorn M, Guha P, Cunetta M, Espat NJ, Miller G, Junghans RP (2016). Tumor-associated GM-CSF overexpression induces immunoinhibitory molecules via STAT3 in myeloid-suppressor cells infiltrating liver metastases. Cancer Gene Ther.

[87] Guha P, Gardell J, Darpolor J, Cunetta M, Lima M, Miller G (2019). STAT3 inhibition induces Bax-dependent apoptosis in liver tumor myeloid-derived suppressor cells. Oncogene.

[88] Chiu DK, Tse AP, Xu IM, Di Cui J, Lai RK, Li LL (2017). Hypoxia inducible factor HIF-1 promotes myeloid-derived suppressor cells accumulation through ENTPD2/CD39L1 in hepatocellular carcinoma. Nat Commun.

[89] Noman MZ, Desantis G, Janji B, Hasmim M, Karray S, Dessen P (2014). PD-L1 is a novel direct target of HIF-1α, and its blockade under hypoxia enhanced MDSC-mediated T cell activation. J Exp Med.

[90] He Q, Liu M, Huang W, Chen X, Zhang B, Zhang T (2021). IL-1β-induced elevation of solute carrier family 7 member 11 promotes hepatocellular carcinoma metastasis through up-regulating programmed death ligand 1 and colony-stimulating factor 1. Hepatology.

[91] Kwong TT, Wong CH, Zhou JY, Cheng ASL, Sung JJY, Chan AWH (2020). Chemotherapy-induced recruitment of myeloid-derived suppressor cells abrogates efficacy of immune checkpoint blockade. JHEP Rep..

[92] Guo W, Zhang Q, Du Y, Guo J, Zhao T, Bai L, An X (2021). Immunomodulatory activity of polysaccharides from *Brassica*
*rapa* by activating Akt/NF-κB signaling. Chin Herb Med.

[93] Lin L, Chen S, Wang H, Gao B, Kallakury B, Bhuvaneshwar K (2021). SPTBN1 inhibits inflammatory responses and hepatocarcinogenesis via the stabilization of SOCS1 and downregulation of p65 in hepatocellular carcinoma. Theranostics.

[94] Xia S, Wu J, Zhou W, Zhang M, Zhao K, Liu J (2021). SLC7A2 deficiency promotes hepatocellular carcinoma progression by enhancing recruitment of myeloid-derived suppressors cells. Cell Death Dis.

[95] Wang D, Li X, Li J, Lu Y, Zhao S, Tang X (2019). APOBEC3B interaction with PRC2 modulates microenvironment to promote HCC progression. Gut.

[96] Porta C, Consonni FM, Morlacchi S, Sangaletti S, Bleve A, Totaro MG (2020). Tumor-derived prostaglandin E2 promotes p50 NF-κB-dependent differentiation of monocytic MDSCs. Cancer Res.

[97] Xia L, Oyang L, Lin J, Tan S, Han Y, Wu N (2021). The cancer metabolic reprogramming and immune response. Mol Cancer.

[98] Mondanelli G, Bianchi R, Pallotta MT, Orabona C, Albini E, Iacono A (2017). A relay pathway between arginine and tryptophan metabolism confers immunosuppressive properties on dendritic cells. Immunity.

[99] Lin Y, Cai Q, Chen Y, Shi T, Liu W, Mao L (2022). CAFs shape myeloid-derived suppressor cells to promote stemness of intrahepatic cholangiocarcinoma through 5-lipoxygenase. Hepatology.

[100] Zhao H, Teng D, Yang L, Xu X, Chen J, Jiang T (2022). Myeloid-derived itaconate suppresses cytotoxic CD8+ T cells and promotes tumour growth. Nat Metab.

[101] Zhang Q, Ma C, Duan Y, Heinrich B, Rosato U, Diggs LP (2021). Gut microbiome directs hepatocytes to recruit MDSCs and promote cholangiocarcinoma. Cancer Discov.

[102] Schneider KM, Mohs A, Gui W, Galvez EJC, Candels LS, Hoenicke L (2022). Imbalanced gut microbiota fuels hepatocellular carcinoma development by shaping the hepatic inflammatory microenvironment. Nat Commun.

[103] Halaby MJ, Hezaveh K, Lamorte S, Ciudad MT, Kloetgen A, MacLeod BL (2019). GCN2 drives macrophage and MDSC function and immunosuppression in the tumor microenvironment. Sci Immunol..

[104] Kim R, Hashimoto A, Markosyan N, Tyurin VA, Tyurina YY, Kar G, Fu S (2022). Ferroptosis of tumour neutrophils causes immune suppression in cancer. Nature.

[105] Wang T, Hu Y, Dusi S, Qi F, Sartoris S, Ugel S (2023). "Open Sesame" to the complexity of pattern recognition receptors of myeloid-derived suppressor cells in cancer. Front Immunol.

[106] Liu H, Ling CC, Yeung WHO, Pang L, Liu J, Zhou J (2021). Monocytic MDSC mobilization promotes tumor recurrence after liver transplantation via CXCL10/TLR4/MMP14 signaling. Cell Death Dis.

[107] Maisonneuve C, Tsang DKL, Foerster EG, Robert LM, Mukherjee T, Prescott D (2021). Nod1 promotes colorectal carcinogenesis by regulating the immunosuppressive functions of tumor-infiltrating myeloid cells. Cell Rep.

[108] Johnson LR, Lee DY, Eacret JS, Ye D, June CH, Minn AJ (2021). The immunostimulatory RNA RN7SL1 enables CAR-T cells to enhance autonomous and endogenous immune function. Cell.

[109] Zeng X, Liao G, Li S, Liu H, Zhao X, Li S (2023). Eliminating METTL1-mediated accumulation of PMN-MDSCs prevents hepatocellular carcinoma recurrence after radiofrequency ablation. Hepatology.

[110] Liu M, Zhou J, Liu X, Feng Y, Yang W, Wu F (2020). Targeting monocyte-intrinsic enhancer reprogramming improves immunotherapy efficacy in hepatocellular carcinoma. Gut.

[111] Liu BQ, Bao ZY, Zhu JY, Liu H (2021). Fibrinogen-like protein 2 promotes the accumulation of myeloid-derived suppressor cells in the hepatocellular carcinoma tumor microenvironment. Oncol Lett.

[112] Haverkamp JM, Smith AM, Weinlich R, Dillon CP, Qualls JE, Neale G (2014). Myeloid-derived suppressor activity is mediated by monocytic lineages maintained by continuous inhibition of extrinsic and intrinsic death pathways. Immunity.

[113] Fiore A, Ugel S, De Sanctis F, Sandri S, Fracasso G, Trovato R (2018). Induction of immunosuppressive functions and NF-κB by FLIP in monocytes. Nat Commun.

[114] Alshetaiwi H, Pervolarakis N, McIntyre LL, Ma D, Nguyen Q, Rath JA (2020). Defining the emergence of myeloid-derived suppressor cells in breast cancer using single-cell transcriptomics. Sci Immunol..

[115] Deng LJ, Qi M, Li N, Lei YH, Zhang DM, Chen JX (2020). Natural products and their derivatives: promising modulators of tumor immunotherapy. J Leukoc Biol.

[116] Wang Y, Zhang Q, Chen Y, Liang CL, Liu H, Qiu F (2020). Antitumor effects of immunity-enhancing traditional Chinese medicine. Biomed Pharmacother.

[117] Zhang Y, Lou Y, Wang J, Yu C, Shen W (2021). Research status and molecular mechanism of the traditional Chinese medicine and antitumor therapy combined strategy based on tumor microenvironment. Front Immunol.

[118] Tu SP, Jin H, Shi JD, Zhu LM, Suo Y, Lu G (2012). Curcumin induces the differentiation of myeloid-derived suppressor cells and inhibits their interaction with cancer cells and related tumor growth. Cancer Prev Res (Phila).

[119] Tian S, Liao L, Zhou Q, Huang X, Zheng P, Guo Y (2021). Curcumin inhibits the growth of liver cancer by impairing myeloid-derived suppressor cells in murine tumor tissues. Oncol Lett.

[120] Tao H, Liu M, Wang Y, Luo S, Xu Y, Ye B (2021). Icaritin induces anti-tumor immune responses in hepatocellular carcinoma by inhibiting splenic myeloid-derived suppressor cell generation. Front Immunol.

[121] Kim K, Yang WH, Jung YS, Cha JH (2020). A new aspect of an old friend: the beneficial effect of metformin on anti-tumor immunity. BMB Rep.

[122] Eikawa S, Nishida M, Mizukami S, Yamazaki C, Nakayama E, Udono H (2015). Immune-mediated antitumor effect by type 2 diabetes drug, metformin. Proc Natl Acad Sci USA.

[123] Baumann T, Dunkel A, Schmid C, Schmitt S, Hiltensperger M, Lohr K (2020). Regulatory myeloid cells paralyze T cells through cell-cell transfer of the metabolite methylglyoxal. Nat Immunol.

[124] Li TY, Yang Y, Zhou G, Tu ZK (2019). Immune suppression in chronic hepatitis B infection associated liver disease: a review. World J Gastroenterol.

[125] Tsai CL, Chang JS, Yu MC, Lee CH, Chen TC, Chuang WY, Kuo WL, Lin CC, Lin SM, Hsieh SY. Functional genomics identifies hepatitis-induced STAT3-TYRO3-STAT3 signaling as a potential therapeutic target of hepatoma. Clin Cancer Res. 2020;26(5):1185-97. 10.1158/1078-0432.CCR-18-3531. 10.1158/1078-0432.CCR-18-3531 31831556

[126] Williams M, Liu X, Zhang Y, Reske J, Bahal D, Gohl TG (2020). NCOA5 deficiency promotes a unique liver protumorigenic microenvironment through p21WAF1/CIP1 overexpression, which is reversed by metformin. Oncogene.

[127] Voron T, Marcheteau E, Pernot S, Colussi O, Tartour E, Taieb J, Terme M (2014). Control of the immune response by pro-angiogenic factors. Front Oncol.

[128] Shojaei F, Wu X, Malik AK, Zhong C, Baldwin ME, Schanz S (2007). Tumor refractoriness to anti-VEGF treatment is mediated by CD11b+Gr1+ myeloid cells. Nat Biotechnol.

[129] Chen Y, Huang Y, Reiberger T, Duyverman AM, Huang P, Samuel R (2014). Differential effects of sorafenib on liver versus tumor fibrosis mediated by stromal-derived factor 1 alpha/C-X-C receptor type 4 axis and myeloid differentiation antigen-positive myeloid cell infiltration in mice. Hepatology.

[130] Chang CJ, Yang YH, Chiu CJ, Lu LC, Liao CC, Liang CW (2018). Targeting tumor-infiltrating Ly6G+ myeloid cells improves sorafenib efficacy in mouse orthotopic hepatocellular carcinoma. Int J Cancer.

[131] Yang Y, Sun M, Yao W, Wang F, Li X, Wang W (2020). Compound kushen injection relieves tumor-associated macrophage-mediated immunosuppression through TNFR1 and sensitizes hepatocellular carcinoma to sorafenib. J Immunother Cancer.

[132] Holmgaard RB, Zamarin D, Li Y, Gasmi B, Munn DH, Allison JP (2015). Tumor-expressed IDO recruits and activates MDSCs in a Treg-dependent manner. Cell Rep.

[133] Yang X, Lam W, Jiang Z, Guan F, Han X, Hu R (2021). YIV-906 potentiated anti-PD1 action against hepatocellular carcinoma by enhancing adaptive and innate immunity in the tumor microenvironment. Sci Rep.

[134] Darden DB, Bacher R, Brusko MA, Knight P, Hawkins RB, Cox MC (2021). Single-cell RNA-seq of human myeloid-derived suppressor cells in late sepsis reveals multiple subsets with unique transcriptional responses: a pilot study. Shock.

[135] Li X, Li S, Wu B, Xu Q, Teng D, Yang T (2022). Landscape of immune cells heterogeneity in liver transplantation by single-cell RNA sequencing analysis. Front Immunol.

[136] Akiyama T, Yasuda T, Uchihara T, Yasuda-Yoshihara N, Tan BJY, Yonemura A (2023). Stromal reprogramming through dual PDGFRα/β blockade boosts the efficacy of anti-PD-1 immunotherapy in fibrotic tumors. Cancer Res.

[137] Aaboe Jørgensen M, Ugel S, Linder Hübbe M, Carretta M, Perez-Penco M, Weis-Banke SE (2021). Arginase 1-based immune modulatory vaccines induce anticancer immunity and synergize with anti-PD-1 checkpoint blockade. Cancer Immunol Res.

[138] Canè S, Barouni RM, Fabbi M, Cuozzo J, Fracasso G, Adamo A (2023). Neutralization of NET-associated human ARG1 enhances cancer immunotherapy. Sci Transl Med..

[139] Wilkerson A, Kim J, Huang AY, Zhang M (2017). Nanoparticle systems modulating myeloid-derived suppressor cells for cancer immunotherapy. Curr Top Med Chem.

[140] Ding D, Zhong H, Liang R, Lan T, Zhu X, Huang S (2021). Multifunctional nanodrug mediates synergistic photodynamic therapy and MDSCs-targeting immunotherapy of colon cancer. Adv Sci (Weinh)..

[141] Hegde S, Leader AM, Merad M (2021). MDSC: Markers, development, states, and unaddressed complexity. Immunity.

[142] Vanhaver C, Bruggen P, Bruger AM (2021). MDSC in mice and men: mechanisms of immunosuppression in Cancer. J Clin Med.

[143] Yaseen MM, Abuharfeil NM, Darmani H, Daoud A (2021). Recent advances in myeloid-derived suppressor cell biology. Front Med.

[144] Duya PA, Chen Y, Bai L, Li Z, Li J, Chai R (2022). Nature products of traditional Chinese medicine provide new ideas in γδT cell for tumor immunotherapy. Acupuncture and Herbal Medicine.

